# Whole-genome sequencing for surveillance of *Salmonella* at a public health institution in South Africa

**DOI:** 10.4102/ajlm.v14i1.2900

**Published:** 2025-12-09

**Authors:** Anthony M. Smith, Phuti Sekwadi, Hlengiwe M. Ngomane, Bolele Disenyeng, Linda K. Erasmus, Juno Thomas, Dineo Bogoshi, Shannon L. Smouse, Nomsa P. Tau

**Affiliations:** 1 Centre for Enteric Diseases, National Institute for Communicable Diseases, Johannesburg, South Africa; 2 Department of Medical Microbiology, Faculty of Health Sciences, University of Pretoria, Pretoria, South Africa

**Keywords:** Salmonella, whole-genome sequencing, genomics, surveillance, outbreak, cluster, South Africa, Africa, public health

## Abstract

**Background:**

Whole-genome sequencing (WGS) is transforming communicable disease surveillance globally. The National Institute for Communicable Diseases, South Africa, participates in national laboratory-based surveillance for human isolates of *Salmonella*.

**Objective:**

This study was to investigate human *Salmonella* isolates from South Africa, 2020–2023, using WGS analysis.

**Methods:**

WGS was performed using Illumina NextSeq Technology. Data were analysed using multiple bioinformatics tools, including those available at the Center for Genomic Epidemiology, Pathogenwatch and EnteroBase. Data analysis allowed for identification and characterisation of isolates. Core-genome multilocus sequence typing was used to investigate the phylogeny of isolates.

**Results:**

Of the 8006 isolates of *Salmonella* that were analysed using WGS, 130 distinctive serovars and subspecies were identified. *Salmonella enterica* serovar Enteritidis (*Salmonella* Enteritidis) (4271/8006; 53.3%) and *Salmonella* Typhimurium (1430/8006; 17.9%) were the most prevalent serovars, accounting for 71.2% of all isolates. This was followed by *Salmonella* Typhi (482/8006; 6.0%). Sixteen per cent (1288/8006) of isolates showed the presence of antimicrobial resistance (AMR) determinants associated with ≥ 2 classes of antimicrobials. *Salmonella* Isangi (167/8006; 2.1%) showed the highest prevalence of AMR, with most isolates (159/167; 95.2%) showing AMR determinants associated with ≥ 7 classes of antimicrobials. Core-genome multilocus sequence typing was used to confirm several suspected clusters and outbreaks and identified additional cryptic or unreported clusters and outbreaks. Investigation of clusters and outbreaks mostly involved *Salmonella* Enteritidis and *Salmonella* Typhi.

**Conclusion:**

The implementation of WGS has enabled genomic surveillance of *Salmonella*, which allows for enhanced characterisation and AMR determination of isolates and identification of clusters and outbreaks, which informs targeted public health investigation and response.

**What this study adds:**

This study describes the population structure of *Salmonella* isolated from humans in South Africa and hugely contributes to the available *Salmonella* WGS data from Africa.

## Introduction

*Salmonella* remains a major cause of human disease worldwide, particularly in developing countries, where they are a leading cause of morbidity and mortality.^1,2^ In Africa, *Salmonella* disease is largely associated with non-invasive gastrointestinal infections; however, a sizable amount of the disease is also caused by invasive infections. In Africa, invasive *Salmonella* infections are associated with both typhoidal and non-typhoidal *Salmonella*.^3,4^ Surveillance and laboratory characterisation of *Salmonella* is important to monitor prevalence and trends in *Salmonella* infections. Recent trends in public health microbiology have shown an evolution towards whole-genome sequencing (WGS) as the methodology of choice for laboratory investigation of infectious diseases. Globally, many public health institutions have transitioned to WGS as their primary methodology for characterisation of bacterial pathogens, and this has included *Salmonella*.^5,6,7^ The World Health Organization has endorsed genomics and WGS approaches to investigate various communicable diseases.^8,9^

The National Institute for Communicable Diseases (NICD) (https://www.nicd.ac.za/) is a national public health institute for South Africa, providing disease surveillance, specialised diagnostic services, outbreak response, public health research, and capacity building to support the government’s response to communicable disease threats. The Centre for Enteric Diseases (CED), NICD, performs surveillance on pathogens associated with diarrhoea and enteric fever, and is involved with investigation and response to enteric disease outbreaks. The CED also provides specialised reference laboratory testing for enteric bacteria and viruses. In addition, the CED plays a part in national laboratory-based surveillance for human isolates of *Salmonella*,^10^ whereby isolates of *Salmonella* are received from more than 200 public and private clinical microbiology laboratories throughout South Africa. Suspected or laboratory-confirmed cases of enteric fever and clinical laboratory identifications of *Salmonella* isolates are ‘notifiable medical conditions’ in South Africa and it is thus mandatory for these to be reported to the Department of Health.^11^ The CED performs routine WGS on all *Salmonella* isolates received. Whole-genome sequencing data are analysed to confirm the identification of isolates with respect to genus, species and serovar, and further characterise the isolates with respect to multilocus sequence typing (MLST) and presence of antimicrobial resistance (AMR) determinants. Furthermore, WGS data is also submitted to the public EnteroBase platform (https://enterobase.warwick.ac.uk/species/index/senterica),^12^ where data are further interrogated using core-genome MLST (cgMLST) to investigate for clusters of genetically related cases (predictive of possible outbreaks) and to complement epidemiological investigation of outbreaks. Before implementation of WGS, presumptive *Salmonella* isolates were identified using traditional phenotypic microbiological methods, which included VITEK identification and serotyping performed according to the White-Kauffmann-Le Minor Scheme.^13,14^ This would have been followed by molecular subtyping of isolates, on selected isolates only (mostly associated with outbreak investigations), using methods such as pulsed-field gel electrophoresis analysis and multiple-locus variable-number tandem-repeats analysis.^13,14,15,16^

In 2020, CED implemented WGS for routine surveillance of clinical isolates of *Salmonella*. We now present the results of this WGS implementation and summarise key findings following analysis of isolates from 2020 to 2023. We report on the number of isolates sequenced, predominant serovars and subspecies identified, significant strains identified, notable AMR profiles identified, clusters identified, and outbreaks investigated.

## Methods

### Ethical considerations

Ethical approval to perform surveillance activities and laboratory analysis on clinical isolates of *Salmonella* was obtained from the Human Research Ethics Committee of the University of the Witwatersrand, Johannesburg, South Africa (protocol reference numbers: M160667, M1809107, M210752, M230985). Databases where patient data are stored are password protected and the patient identifiers were removed from genomic data shared at public repositories.

### Surveillance for clinical isolates of *Salmonella* in South Africa

This project started on 01 January 2020 and ended on 31 December 2023. The NICD is a national public health institute for South Africa, providing disease surveillance, specialised diagnostic services, outbreak response, public health research, and capacity building to support the government’s response to communicable disease threats. The CED plays a part in national laboratory-based surveillance for human isolates of *Salmonella*. Isolates were received from more than 200 public and private clinical microbiology laboratories throughout the country. After *Salmonella* identification at laboratories, isolates were usually received at the CED within 1–4 weeks. Following receipt at the CED, isolates were immediately processed for WGS analysis (as described below), a process which is usually completed within 2–3 weeks.

### Metadata and epidemiological investigation

*Salmonella* isolates were received with information related to basic metadata, including details of the patient, place of residence and specimen collection date; data all obtained from laboratory request forms. In some situations, such as cases of enteric fever, cases associated with outbreak investigations and cases from some enhanced surveillance sites; patients were followed up to obtain more detailed information and case investigation forms were completed. Clinical laboratory identifications of *Salmonella* isolates are ‘notifiable medical conditions’ in South Africa, so it is mandatory for these to be reported to the Department of Health.

### Receipt of bacterial cultures and phenotypic characterisation

The CED received and processed isolates using methodology as previously described.^17,18^ Methodology is briefly described as follows. Following receipt of presumptive isolates on Dorset-Egg transport media (Diagnostic Media Products, National Health Laboratory Service, Johannesburg, South Africa), isolates are sub-cultured onto 5% Blood Agar (Diagnostic Media Products) to check for viability and purity, following which the isolates are processed to extract genomic DNA for WGS analysis. If there is suspicion that a culture is not a *Salmonella*, then that culture will be further investigated using standard phenotypic microbiological identification and serotyping methodologies, including VITEK-2 identification (bioMérieux, Marcy-l’Étoile, France) and serotyping completed as per the White-Kauffmann-Le Minor Scheme. When required, antimicrobial (ampicillin, ciprofloxacin, ceftriaxone, azithromycin) susceptibility testing was achieved via the Etest methodology (bioMérieux). Interpretation of susceptibility data was performed as per the Clinical and Laboratory Standards Institute guidelines.^19^

### Genomic DNA extraction and whole-genome sequencing of bacteria

Genomic DNA was extracted from bacteria using either the Qiagen QIAamp DNA Mini Kit (QIAGEN, Hilden, Germany) or the Invitrogen PureLink Microbiome DNA Purification Kit (Invitrogen, Waltham, Massachusetts, United States). Whole-genome sequencing was performed by the NICD Sequencing Core Facility (SCF). From 2020 to 2023, WGS was performed using various models of Illumina equipment (Illumina, San Diego, California, United States), including Illumina MiSeq, Illumina NextSeq 550 and Illumina NextSeq 1000. DNA libraries were prepared using various Illumina kits, including the Nextera XT DNA Library Preparation Kit, the Nextera DNA Flex Library Preparation Kit and the Illumina DNA Prep Kit. Sequencing included paired-end sequencing runs, including ~80 times coverage.

### Analysis of whole-genome sequencing data

The CED performed analysis of WGS data using methodology as previously described.^17,18^ Methodology is briefly described as follows. Illumina data were processed and investigated with the JEKESA bioinformatics pipeline (https://github.com/stanikae/jekesa), which includes several analysis tools. Default options were set for all tools, unless otherwise mentioned. Quality control and filtering of reads were performed with FastQC version 0.11.9 (https://www.bioinformatics.babraham.ac.uk/projects/fastqc/) and TrimGalore version 0.6.2 set at a minimum Phred quality score of 30 and minimum read length of 50 bp. Identification of species and detection to closest reference were accomplished using BactInspector version 0.1.3 (https://gitlab.com/antunderwood/bactinspector). Checking for contamination was accomplished with ConFindr version 0.7.4 (https://github.com/OLC-Bioinformatics/ConFindr) and Kraken2 version 2.0.8-beta (https://github.com/DerrickWood/kraken2/releases). *De novo* assembly was accomplished using SKESA version 2.3.0 (https://github.com/ncbi/SKESA), followed by optimisation of the assemblies with Shovill version 1.1.0 (https://github.com/tseemann/shovill), with depth set to 100 and minimum contig length set to 200. Assessment of assembly metrics were performed with QUAST version 5.0.2 using PubMLST typing schemes (https://pubmlst.org/). Identification of AMR determinants was accomplished with ResFinder version 4.1 (https://www.genomicepidemiology.org/services/) and NCBI AMRfinder version 3.11.26.^20^ Prediction of *Salmonella* serovars was accomplished with SeqSero2 version 1.1.0 (https://denglab.info/SeqSero2) and SISTR version 1.1.2 (https://github.com/phac-nml/sistr_cmd).

Investigation of WGS data was further accomplished at EnteroBase (http://enterobase.warwick.ac.uk/species/index/senterica). Raw sequencing data were submitted to EnteroBase, where the data were quality checked, assembled and analysed via multiple tools, to provide information concerning *Salmonella* serovar, AMR determinants, MLST and cgMLST. The phylogeny of isolates was explored with the EnteroBase cgMLST tool incorporating the ‘cgMLST V2 + HierCC V1’ scheme, which performs an analysis on 3002 core genes of *Salmonella*. The phylogeny and genetic relatedness of isolates were visualised with a GrapeTree-generated minimum spanning tree using the ‘MSTree V2’ algorithm.^21^ For cluster detection, we followed the following steps. Once a GrapeTree was produced, the settings/operators of the tool were set to ‘collapse branches’ at a value of ‘5’, which ensued that isolates showing ≤ 5 allelic differences were collapsed together into a ‘cluster’. Our cluster definition was ≥ 3 isolates showing ≤ 5 allelic differences, as found by the above actions, following cgMLST analysis and creation of a GrapeTree. For all *Salmonella* serovars (except *S. enterica* serovar Enteritidis [*Salmonella* Enteritidis]), we defined a cluster of isolates at a ≤ 5-allele difference threshold, to denote high genetic relatedness among isolates and identify cases likely associated with a common cause (epidemiological link). *Salmonella* Enteritidis is a highly clonal serovar, so in order to refine cluster identification for this serovar to obtain the most epidemiological informative clusters, we lowered the cluster definition threshold to a 0-allele difference. Clusters were assigned (associated with) EnteroBase cgMLST hierarchical cluster level 5 identifying numbers (hierarchical cluster level 5 is where isolates are clustered at five allele differences).

### Data availability

Sequencing data were uploaded to the public EnteroBase platform (http://enterobase.warwick.ac.uk/species/index/senterica) and are freely available to access at this platform. Data are also available at the European Nucleotide Archive under the project accession numbers PRJEB39002, PRJEB39546, and PRJEB39988.

## Results

### Turnaround time to whole-genome sequencing results and costing of whole-genome sequencing

Following genomic DNA extraction from bacteria, we typically batch samples and submit weekly (usually on a Friday) to our SCF. The turnaround time to completion of WGS at our SCF is ~10 working days. For urgent sequencing, such as for outbreak investigations, sequencing can be fast-tracked, resulting in decreased turnaround times (3–5 working days). In general, for routine sequencing, the turnaround time from receipt of a culture at the CED laboratory to completion of analysis of WGS data, is ~15 working days.

The costs to perform Illumina WGS have steadily decreased year on year. In January 2020, our cost to perform Illumina WGS (paired-end sequencing, at ~80 times coverage) on a single Salmonella isolate was ~R2510 South African Rand (ZAR) as compared to ~R1210 ZAR in December 2023 (R0.055 ZAR to United States dollar conversion rate, on 10 March 2025). So, WGS has become more affordable with time.

### Sharing of whole-genome sequencing data and public health action

All WGS data were uploaded and shared at the EnteroBase *Salmonella* database (http://enterobase.warwick.ac.uk/species/index/senterica). Data shared at EnteroBase are immediately made available publicly to benefit the global public health and research community. EnteroBase also auto-uploads data to the Sequence Read Archive, following which project and sample accession numbers are assigned to isolate data. As of 10 March, 2025, EnteroBase ranked South Africa as country number one with respect to the number of *Salmonella* genome submissions from Africa, and seventh with respect to global country submissions.

As required, *Salmonella* WGS data was presented and discussed at the NICD weekly ‘Communicable Diseases Meetings’. These meetings include representatives from the NICD Outbreak Response Unit, all NICD Centres, and epidemiologists from all provinces across South Africa. Matters discussed included: any interesting findings related to routine disease surveillance activities, trends in disease notifications and reporting, reports of disease clusters, outbreak investigations, and new/emerging cases of diseases. Centre for Enteric Disease will report on any significant findings related to analysis of *Salmonella* WGS data, including clusters identified and outbreak investigations. As required, representatives of our Outbreak Response Unit will communicate with our Department of Health on all relevant matters. So, the chain of custody for reporting WGS data for public health action is: NICD Centre > NICD Outbreak Response Unit > Department of Health > public notification (as required), and further sharing of information.

### Top (most common) eight *Salmonella* serovars and subspecies in South Africa

**FIGURE 1:**
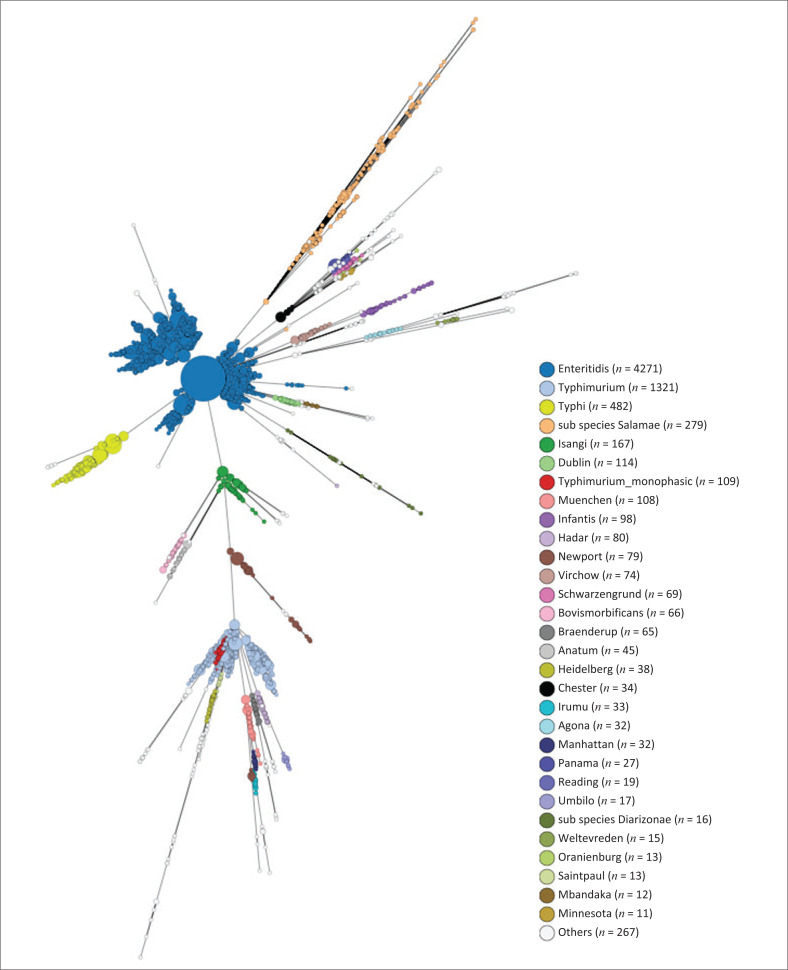
Minimum spanning tree created using cgMLST data for *Salmonella* isolates (*N* = 8006), South Africa, 2020–2023. The spherical nodes represent isolates. The larger the spherical node, the more isolates which are indicated. The figure legend lists *Salmonella* serovars and subspecies identified, in order from highest to lowest number of isolates. cgMLST, core-genome multilocus sequencing typing.

**FIGURE 2:**
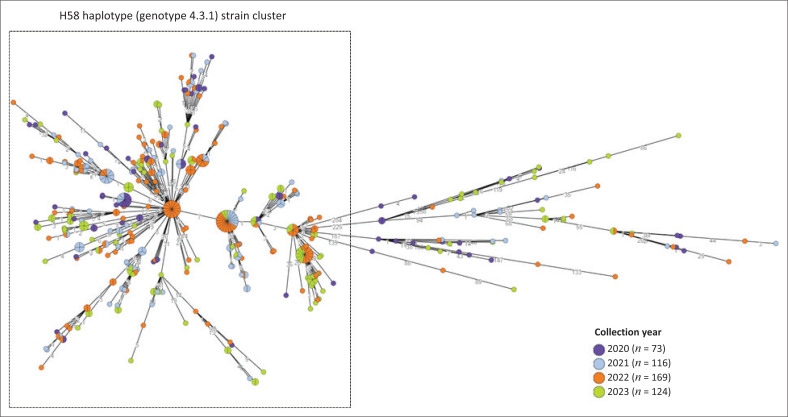
Minimum spanning tree created using cgMLST data for *Salmonella* Typhi isolates (*N* = 482), South Africa, 2020–2023. The spherical nodes represent isolates. The larger the spherical node, the more isolates which are indicated. The number of segments within a spherical node is representative of the number of isolates. The number values between adjoining nodes specify the number of allele differences between connecting nodes (isolates). The figure legend lists the year of isolation. The cluster of H58 haplotype (genotype 4.3.1) strains is indicated. cgMLST, core-genome multilocus sequencing typing.

From 2020 to 2023, 8006 isolates of *Salmonella* were analysed using WGS. One hundred and thirty distinctive *Salmonella* serovars or subspecies were identified (Figure 1). *Salmonella* Enteritidis (4271/8006; 53.3%) and *Salmonella* Typhimurium (1430/8006; 17.9%) were the most prevalent serovars, accounting for 71.2% of all isolates. The following serovars or subspecies completed our top (most common) eight: *Salmonella* Typhi (482/8006; 6.0%), *S. enterica* subspecies *salamae* (279/8006; 3.5%), *Salmonella* Isangi (167/8006; 2.1%), *Salmonella* Dublin (114/8006; 1.4%), *Salmonella* Muenchen (108/8006; 1.3%), and *Salmonella* Infantis (98/8006; 1.2%). For *Salmonella* Typhi, 414/482 (85.9%) were of the H58 haplotype (genotype 4.3.1) strain (Figure 2). For *Salmonella* Typhimurium, 269/1430 (18.8%) isolates were of the ST313 variant; while 109/1430 (7.6%) isolates were of the monophasic variant (1,4,[5],12:i:-).

### Notable clusters and outbreak investigations

**FIGURE 3:**
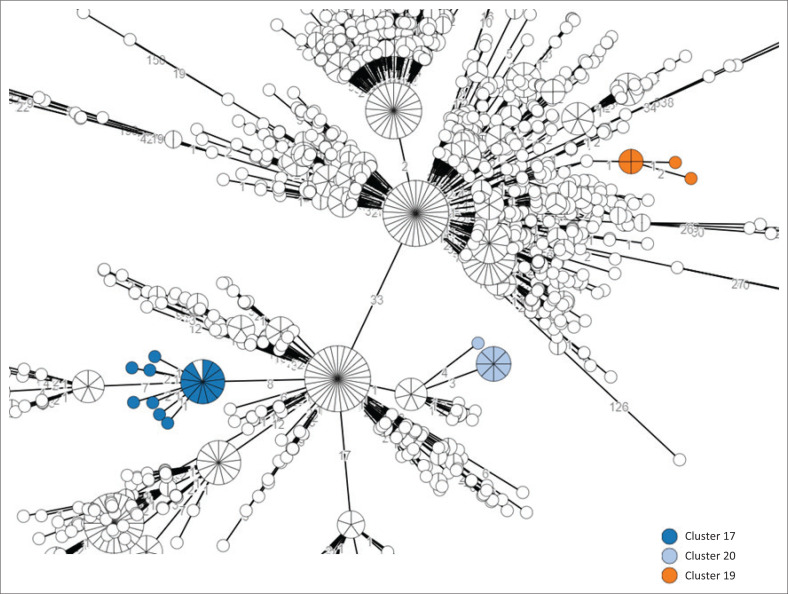
Snapshot from a minimum spanning tree created using cgMLST data for *Salmonella* Enteritidis isolates, South Africa, 2020–2023. The spherical nodes represent isolates. The larger the spherical node, the more isolates which are indicated. The number of segments within a spherical node is representative of the number of isolates. The number values between adjoining nodes specify the number of allele differences between connecting nodes (isolates). The figure legend points to some notable clusters investigated, of which details are described in Table 1. cgMLST, core-genome multilocus sequencing typing.

**FIGURE 4:**
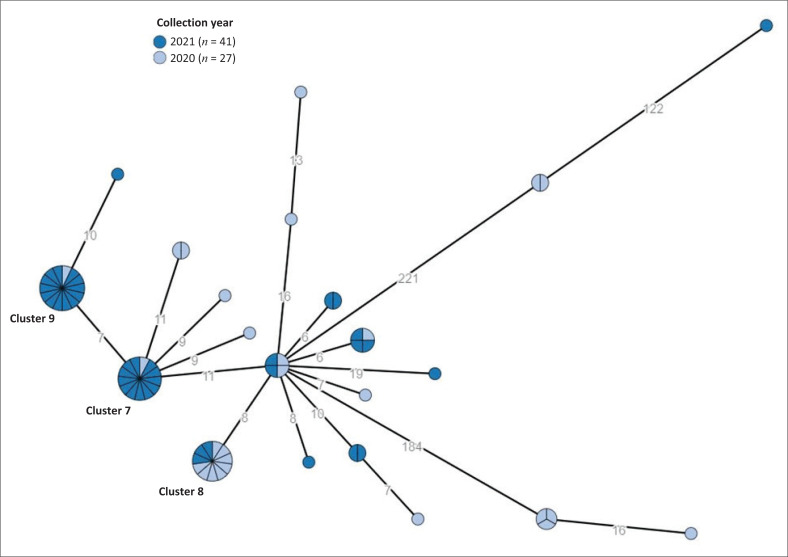
Minimum spanning tree created using cgMLST data for *Salmonella* Typhi isolates (*N* = 68) sourced from the Western Cape province of South Africa, 2020–2021. The spherical nodes represent isolates. Isolates showing ≤ 5 allelic differences, are collapsed together into a single spherical node. The larger the spherical node, the more isolates which are indicated. The number of segments within a spherical node is representative of the number of isolates. The number values between adjoining nodes specify the number of allele differences between connecting nodes (isolates). The figure legend lists the year of isolation. Some notable clusters investigated are indicated, of which details are described in Table 1. cgMLST, core-genome multilocus sequencing typing.

Table 1 provides a summary of notable *Salmonella* clusters and outbreaks investigated in South Africa from 2020 to 2023. These investigations included the following serovars: *Salmonella* Enteritidis, *Salmonella* Typhi, *Salmonella* Typhimurium, *Salmonella* Isangi, *Salmonella* Panama, *Salmonella* Vejle, *Salmonella* Newport, and *Salmonella* Muenchen. Most investigations were associated with *Salmonella* Enteritidis and *Salmonella* Typhi. Figure 3 shows some clusters investigated within the background of other circulating isolates, for *Salmonella* Enteritidis, and Figure 4 shows the same for *Salmonella* Typhi.

**TABLE 1:** Notable *Salmonella* clusters and outbreaks investigated in South Africa, 2020–2023.

Cluster number	*Salmonella* serovar	MLST†	cgMLST HC5 number(s)‡	Date	Province	Suspected items or exposures to have caused the outbreak – possible modes of transmission	Number of cases associated with the outbreak	Number of isolates sequenced	References
1	Enteritidis	11	84774	February 2020	KwaZulu-Natal	Food (chicken meat)	14	6	No reference
2	Enteritidis	11	72007	March 2020	KwaZulu-Natal	Food (chicken meat/pasta)	3	3	No reference
3	Panama	48	236243	June 2020	KwaZulu-Natal	Food (goat meat)	16	16	No reference
4	Vejle	370	245585	September 2020	KwaZulu-Natal	Food (goat meat)	21	5	No reference
5	Typhimurium	19	250745	November 2020	KwaZulu-Natal	Food (cow meat)	46	11	No reference
6	Enteritidis	11	72007	November 2020	KwaZulu-Natal	Unknown	6	6	No reference
7	Typhi	1	202	August 2020 – December 2021	Western Cape	Unknown	13	13	^17^
8	Typhi	1	26478	October 2020 – May 2021	Western Cape	Unknown	11	11	^17^
9	Typhi	1	202	November 2020 – December 2021	Western Cape	Unknown	14	14	^17^
10	Typhi	1	268783, 275473	November 2020 – September 2022	North West, Gauteng, Mpumalanga, Free State, KwaZulu-Natal	Associated with illegal gold miners	53	53	^32^
11	Enteritidis	11	2037	January 2021 – February 2021	Eastern Cape	Unknown	26	26	No reference
12	Enteritidis	11	2037	June 2021	Western Cape	Food (fish)	8	8	No reference
13	Enteritidis	11	2037	September 2021 – October 2021	Western Cape	Food (milk formula)	5	5	No reference
14	Enteritidis	11	2037	November 2021	Gauteng	Food (unknown items)	27	5	No reference
15	Isangi	335	236918	April 2022 – July 2022	Eastern Cape	Nosocomial infection	43	29	^35^
16	Newport	45	313853	May 2022 – July 2022	Western Cape	Unknown	8	8	No reference
17	Enteritidis	11	72007	September 2022	Free State	Food (chicken pasta)	49	19	^43^
18	Muenchen	82	350337	January 2023	KwaZulu-Natal	Unknown	9	3	No reference
19	Enteritidis	11	2037	August 2023	Gauteng	Unknown	24	6	No reference
20	Enteritidis	11	72007	October 2023	KwaZulu-Natal	Unknown	12	9	No reference

Note: Please see the full reference list of this article for details on the articles cited: Smith AM, Sekwadi P, Ngomane HM, et al. Whole-genome sequencing for surveillance of *Salmonella* at a public health institution in South Africa. Afr J Lab Med. 2025;14(1), a2900. https://doi.org/10.4102/ajlm.v14i1.2900.

MLST, multilocus sequencing typing; cgMLST, core-genome MLST; HC5, hierarchical cluster level 5.

† , as per assignment at PubMLST (https://pubmlst.org/);

‡ , as per assignment at EnteroBase (http://enterobase.warwick.ac.uk/species/index/senterica).

### Antimicrobial resistance determinants

**FIGURE 5:**
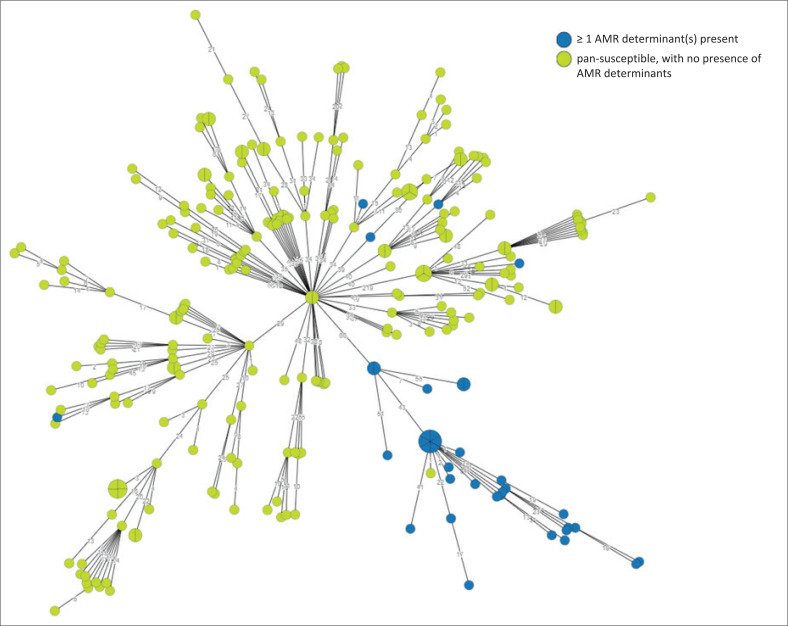
Minimum spanning tree created using cgMLST data for *Salmonella* Typhimurium ST313 isolates (*N* = 269), South Africa, 2020–2023. The circular nodes represent isolates. The larger the circular node, the more isolates which are indicated. The number of segments within a circular node is representative of the number of isolates. The number values between adjoining nodes specify the number of allele differences between connecting nodes (isolates). The legend points to isolates with or without antimicrobial resistance determinants. cgMLST, core-genome multilocus sequencing typing; AMR, antimicrobial resistance.

For AMR determinants, data were reported as per analysis at the EnteroBase *Salmonella* database where the NCBI AMRfinder version 3.11.26 tool^20^ is used to report on the following AMR classes: aminoglycoside, penicillin, extended-spectrum beta-lactamase (ESBL), carbapenemase, colistin, fosfomycin, macrolide, phenicol, quinolone, sulfonamide, tetracycline, and trimethoprim. Sixteen per cent (1288/8006) of isolates showed the presence of AMR determinants associated with ≥ 2 classes of antimicrobials. Among our top (most common) eight serovars or subspecies, *Salmonella* Enteritidis showed the lowest prevalence of AMR, while *Salmonella* Isangi showed the highest prevalence of AMR (Table 2). Most *Salmonella* Isangi (159/167; 95.2%) showed AMR determinants associated with ≥ 7 classes of antimicrobials, including ESBL genes (*bla*_OXA-1_, *bla*_OXA-10_, *bla*_CTX-M-15_, *bla*_TEM-63_, *bla*_DHA_). For *Salmonella* Typhimurium ST313 (*n* = 269), only 37/269 (13.8%) were associated with AMR determinants, while most (232/269; 86.2%) were pan-susceptible (Figure 5).

**TABLE 2:** Antimicrobial resistance determinants associated with the top (most common) eight *Salmonella* serovars and sub-species in South Africa, 2020–2023.

Serovar or subspecies	Isolates associated with ≥ 2 classes of antimicrobials		Isolates associated with ≥ 4 classes of antimicrobials		Isolates associated with ≥ 7 classes of antimicrobials	
	*n*	%	*n*	%	*n*	%
Enteritidis	47/4271	1.1	40/4271	0.9	1/4271	0.02
Subspecies *salamae*	5/279	1.8	2/279	0.7	0/279	0.0
Infantis	11/98	11.2	9/98	9.2	2/98	2.0
Dublin	18/114	15.8	9/114	7.9	1/114	0.9
Muenchen	22/108	20.4	11/108	10.2	0/108	0.0
Typhimurium	457/1430	32.0	226/1430	15.8	28/1430	2.0
Typhi	415/482	86.1	413/482	85.7	20/482	4.1
Isangi	164/167	98.2	164/167	98.2	159/167	95.2

For the period of this research (2020 to 2023), we identified 297 cases of ESBL-positive *Salmonella*, which showed a variety of ESBL genes including: *bla*_CTX-M_ variants, *bla*_CMY_ variants, *bla*_DHA_, *bla*_OXA-1_, *bla*_OXA-10_, *bla*_TEM-63_, and *bla*_SHV-2_. Most ESBL-positive *Salmonella* (162/297; 54.5%) were associated with *Salmonella* Isangi. For carbapenemase-positive *Salmonella*, we identified 22 cases, which included: eight isolates of *Salmonella* Isangi (housing either the *bla*_NDM-1_ gene or *bla*_OXA-181_ gene), four isolates of *Salmonella* Enteritidis (housing either the *bla*_OXA-48_ gene or *bla*_OXA-181_ gene), four isolates of *Salmonella* Typhimurium (housing the *bla*_OXA-48_ gene), two isolates of *Salmonella* Montevideo (housing the *bla*_OXA-48_ gene), one isolate of *Salmonella* Gallinarum (housing the *bla*_OXA-48_ gene), one isolate of *Salmonella* Virchow (housing the *bla*_OXA-48_ gene), one isolate of *Salmonella* Muenster (housing the *bla*_OXA-48_ gene), and one isolate of *S. enterica* subspecies *salamae* (housing the *bla*_OXA-181_ gene).

During this same period of research, we also identified five cases of extensively drug-resistant *Salmonella* Typhi, of which two cases had confirmed travel history to Pakistan. Three isolates included resistome: *bla*_TEM-1_, *bla*_CTX-M-15_, *catA1, sul1, sul2, dfrA7, qnrS1, gyrA* S83F; while two isolates included resistome: *bla*_TEM-1_, *bla*_CTX-M-15_, *catA1, sul1, dfrA7, qnrS1, gyrA* S83F.

## Discussion

The CED, NICD, is a member of the regional PulseNet Africa laboratory network (https://www.pulsenetafrica.org/), which forms part of the PulseNet International network (http://www.pulsenetinternational.org/), a global molecular subtyping network for foodborne disease surveillance. The CED has always followed standardised molecular subtyping methodologies as suggested by PulseNet International, of which in years gone by, the suggested primary methodology was pulsed-field gel electrophoresis analysis. The CED has published extensively on the use of these older (traditional) molecular subtyping methodologies for routine surveillance activities and for investigation of outbreaks involving enteric bacterial pathogens, which have included the use of pulsed-field gel electrophoresis analysis,^15,22^ multiple-locus variable-number tandem-repeats analysis,^14,16^ and MLST (using Sanger sequencing of polymerase chain reaction-amplified genes).^13,14^

In late 2015, CED took the first step towards the use of WGS for analysis of enteric pathogens. This coincided with the establishment of the NICD SCF facility equipped with Illumina MiSeq next-generation sequencing equipment. Our first WGS activities investigated a cluster of *Listeria monocytogenes* cases reported from the Western Cape province, South Africa, 2015. This analysis was timely, as the steering committee of the PulseNet International network was in discussions to start with implementation of WGS, of which the vision of the network for implementation of WGS was later published in 2017.^23^ In 2020, CED terminated the use of all older (traditional) molecular subtyping methodologies (pulsed-field gel electrophoresis and multiple-locus variable-number tandem-repeats analysis), and implemented the use of WGS analysis for routine surveillance and analysis of all clinical isolates of enteric bacterial pathogens, including the *Salmonella*. This was needed in order to align with trends in public health microbiology showing the evolution towards WGS as the primary methodology for laboratory investigation of infectious disease. Globally, many public health institutions and reference laboratories have transitioned to WGS as their primary methodology for characterisation of bacterial pathogens.^5,6,7^

We experienced very few challenges with our implementation of WGS. The reasons probably have been that we implemented well-established and well-validated methodologies, and we were supported by a well-established and well-equipped SCF with dedicated core staff, including bioinformatics support. Whole-genome sequencing all starts with a good quality DNA extraction from bacteria. We used good quality DNA extraction kits to produce quality in our DNA extractions. This then eliminated almost all further problems in downstream sequencing steps, including library preparation. The quality of our sequence data outputs were mostly excellent. On rare occasions, we would encounter assembled data which failed minimum quality thresholds. The quality metrics for our assembled data include an N50 value that must be > 20 kb and number of contigs that must be < 300. For assembled data that fail quality checks, the sample is subjected to a repeated round of sequence analysis, and this usually corrects the quality issue. We rarely encountered contamination problems in our analysis of sequence data. If contamination was encountered, then a repeat DNA extraction on a new pure culture and a new sequence analysis solved the problem. On rare occasions, species identification tools would 100% identify sequence data as a non-*Salmonella* species, and in these situations, presumptive laboratory identifications would be updated to reflect the WGS identification.

Importantly, the implementation of any new laboratory testing methodology must be accompanied by validation data and other checks associated with good laboratory practice. As such, our WGS analysis is accredited as per the international ISO 15189 standard, and we are regularly audited by the South African National Accreditation System, the official laboratory accreditation body of South Africa. Validation of our WGS analysis is continuously affirmed by annual participation in two external WGS Quality Assessment Schemes, one managed by the National Institute for Public Health and the Environment, the Netherlands, in collaboration with the European Centre for Disease Control,^24^ and the other managed by the National Food Institute, Technical University of Denmark.^25^

In the last 5 years, next-generation sequencing technology has advanced rapidly, resulting in shorter turnaround times to WGS results and higher accuracy of sequencing data. In parallel, costs of next-generation sequencing and WGS have also decreased dramatically over recent years, making the technology more affordable and cost-effective for use in public health laboratories. Year-on-year decrease in WGS costs has also been noted by CED, NICD, where the cost to perform Illumina WGS on a single *Salmonella* isolate in January 2020 as compared to the cost in December 2023 decreased by 51.8%. In South Africa, the costs associated with Illumina sequencing is generally more affordable as compared to many other African countries (anecdotal evidence). To some extent, this affordability in South Africa can probably be attributed to the presence of an official Illumina product distributor in the country, namely Separations (https://separations.co.za/). This not only ensures the affordability of reagents and consumables but also ensures rapid order and delivery of said reagents and consumables, timely maintenance and repair of Illumina equipment, and overall good customer support.

Of the 8006 *Salmonella* isolates analysed using WGS, 130 distinctive *Salmonella* serovars and subspecies were identified (Figure 1). *Salmonella* Enteritidis and *Salmonella* Typhimurium (5701/8006; 71.2%) were the most prevalent, which aligns with global trends.^5,26,27^ The following serovars or subspecies completed our top (most common) eight: *Salmonella* Typhi, *S. enterica* subspecies *salamae, Salmonella* Isangi, *Salmonella* Dublin, *Salmonella* Muenchen, and *Salmonella* Infantis. For *Salmonella* Enteritidis, this serovar was associated with the majority of our cluster and outbreak investigations (Table 1), and was mostly associated with a low prevalence of AMR (Table 2). Among *Salmonella* Typhimurium, the ST313 variant was commonly encountered (269/1430; 18.8%). The ST313 variants are known to be highly associated with *Salmonella* bloodstream infections in Africa.^28^ Our reported ST313 variants were mostly (232/269; 86.2%) pan-susceptible (Figure 5), which was an interesting finding, considering that literature reports ST313 variants as typically multidrug-resistant.^28^ For *Salmonella* Typhimurium, 109/1430 (7.6%) of our isolates were of the monophasic variant, of which this variant has emerged globally to become an important pandemic variant with increasing AMR,^27,29^ and increasingly associated with foodborne disease outbreaks.^30^ For *Salmonella* Typhi, most (414/482; 85.9%) of our isolates were of the H58 haplotype (genotype 4.3.1) strain (Figure 2). The H58 haplotype is a globally dominant variant of *Salmonella* Typhi and commonly associated with AMR.^31^ We were able to identify the H58 haplotype using the EnteroBase cgMLST hierarchical cluster assignment tool, where HC50:202 is known to be indicative of the H58 haplotype. Among our *Salmonella* Typhi H58 haplotype strains, most (411/414; 99.3%) showed AMR determinants associated with ≥ 4 classes of antimicrobials, commonly including the following resistome: *bla*_TEM-1B_, *catA1, sul1, sul2, dfrA7. Salmonella* Typhi was often associated with our cluster and outbreak investigations (Table 1). A notable investigation involved *Salmonella* Typhi cases in 2020 to 2022, associated with an outbreak among illegal gold miners, likely resulting from the consumption of contaminated groundwater while working in a gold mine shaft (Table 1).^32^ *Salmonella enterica* subspecies *salamae* (279/8006; 3.5%) was our fourth most prevalent *Salmonella* serovar/subspecies. The *S. enterica* subspecies *salamae* isolates were genetically diverse, with no evidence to suggest any clonal spread (data not shown). Our *S. enterica* subspecies *salamae* data were a surprise finding, considering that they are mostly reported from environmental and animal (mostly cold-blooded animals like reptiles) sources, and are generally considered less pathogenic for humans.^33^ The prevalence of this subspecies in South Africa has previously not been reported. Historically, before the use of WGS analysis, we would have a large contingent of *Salmonella* isolates reported as ‘*Salmonella* species’, because the traditional serotyping methodologies (using antisera) were sometimes inconclusive in making a call on subspecies or serovar. Now with the use of WGS analysis tools, *Salmonella* characterisation is more complete and more accurate, and is now able to more accurately identify *S. enterica* subspecies *salamae*. These isolates were mostly cultured from stool specimens of patients. Unfortunately, no further information was available about these cases, as no follow-up investigations were conducted for these. Also, we are not able to speculate on any possible environmental or zoonotic source for this subspecies, as no further investigations were conducted.

*Salmonella* Isangi (167/8006; 2.1%) was our fifth most prevalent *Salmonella* serovar or subspecies. *Salmonella* Isangi represents an emerging pathogen in South Africa. However, globally, *Salmonella* Isangi is an uncommon serovar. Very few (*n* = 409) *Salmonella* Isangi isolates have been reported in the EnteroBase database (as of 27 March 2025), with most of the cases (203/409; 49.6%) reported from South Africa. Among all the serovars or subspecies in South Africa, *Salmonella* Isangi showed the highest prevalence of AMR (Table 2). Most *Salmonella* Isangi (159/167; 95.2%) showed AMR determinants associated with ≥ 7 classes of antimicrobials, including ESBL genes (*bla*_OXA-1_, *bla*_OXA-10_, *bla*_CTX-M-15_, *bla*_TEM-63_, *bla*_DHA_). Globally (including South Africa), *Salmonella* Isangi are typically multidrug-resistant, and are often associated with hospital outbreaks.^34,35,36,37^ There is a pressing need for studies to identify the reservoir and transmission pathway for *Salmonella* Isangi, as this serotype is very capable of acquiring and retaining extensive drug resistance, and once introduced into the hospital environment, it appears to happily thrive and cause lengthy hospital outbreaks.^35^ *Salmonella* Dublin (114/8006; 1.4%) was our sixth most prevalent *Salmonella* serovar or subspecies. Globally, *Salmonella* Dublin is a relatively uncommon cause of human infections. *Salmonella* Dublin is host-adapted to cattle, so is most prevalent in cattle and cow’s raw milk cheese. Countries that produce large volumes of cheese (such as France) often show an increased prevalence of *Salmonella* Dublin.^38,39^ *Salmonella* Muenchen (108/8006; 1.3%) was our seventh most prevalent *Salmonella* serovar or subspecies. This prevalence aligns with a global reported prevalence of 1.2%, where globally, *Salmonella* Muenchen is listed as the 13th most prevalent *Salmonella* serovar.^27^ *Salmonella* Muenchen is a relatively uncommon cause of human infections globally and there are also very few documented reports of outbreaks associated with *Salmonella* Muenchen. *Salmonella* Infantis (98/8006; 1.2%) was our eighth most prevalent *Salmonella* serovar or subspecies. *Salmonella* Infantis is currently perhaps the biggest mover and shaker among the global *Salmonella* population, gaining increased global prevalence over recent years.^40^ *Salmonella* Infantis has become the fourth most prevalent *Salmonella* serovar causing human infections among European Union member countries.^41^ *Salmonella* Infantis is among the most frequently isolated *Salmonella* serovar in poultry in Europe and the United States.^41^ Globally, *Salmonella* Infantis is currently listed as the third most prevalent *Salmonella* serovar, with a global reported *Salmonella* prevalence of 6.6%.^27^ Interestingly, the population structure of South African *Salmonella* Infantis has been shown to differ substantially from *Salmonella* Infantis isolated elsewhere globally.^42^

### Conclusion

The implementation of WGS for routine surveillance of clinical isolates of *Salmonella* in South Africa has seen a significant increase in the critical mass of *Salmonella* genomic data now available from the African continent. South Africa is currently ranked country number one with respect to the number of *Salmonella* genome submissions from Africa, and seventh with respect to global country submissions. Large WGS data sets, methodically generated over long time periods, provide essential information for: detailed and enhanced characterisation of bacterial strains (pathogens), molecular epidemiological investigations, early detection of clusters of disease, outbreak investigations, investigating for new and emerging strains, investigating for new or unusual AMR profiles, tracking the spread of strains, data for development of treatment strategies and vaccine development, and data for monitoring the effect of treatment interventions and vaccine rollout. Whole-genome sequencing data not only provide value ‘in the now’ but are ‘the gift that keeps on giving’, as data can be further and repeatedly investigated by multiple parties, be that for research purposes or public health activities, all to assist with investigation and containment of future public health threats.
